# Saffold Cardioviruses in Children with Diarrhea, Thailand

**DOI:** 10.3201/eid1706.101983

**Published:** 2011-06

**Authors:** Pattara Khamrin, Natthawan Chaimongkol, Nattika Nantachit, Shoko Okitsu, Hiroshi Ushijima, Niwat Maneekarn

**Affiliations:** Author affiliations: Chiang Mai University, Chiang Mai, Thailand (P. Khamrin, N. Chaimongkol, N. Maneekarn);; Mahidol University, Bangkok, Thailand (N. Nantachit);; Nihon University School of Medicine, Tokyo, Japan (S. Okitsu, H. Ushijima)

**Keywords:** Saffold virus, viruses, cardiovirus, children, diarrhea, Thailand, letter

**To the Editor:** Cardioviruses currently consist of at least 3 viruses: Theiler murine encephalomyocarditis virus, encephalomyocarditis virus, and Saffold virus (SAFV) (*1**–**4*). Saffold cardiovirus in the family *Picornaviridae* was isolated and identified from fecal specimens of a child with fever of unknown origin in the United States (*3*).

Several reports have documented the presence of SAFV in fecal samples and respiratory secretions (*5**–**10*). However, it is not clear whether SAFV is associated with any disease, including gastroenteritis in humans, and epidemiologic data for SAFV are limited. We report an epidemiologic survey of SAFV in children hospitalized with diarrhea in Chiang Mai, Thailand.

A total of 150 fecal specimens were obtained from children hospitalized with acute gastroenteritis in Chiang Mai during January–December 2007. Patient ages ranged from >1 to 5 years. SAFV in fecal specimens was detected by using a nested PCR and primers specific for the virus 5′ untranslated region (*7*). A negative control was also included to monitor any contamination that might have occurred during the PCR.

SAFVs detected were further analyzed by amplification of the viral protein (VP) 1 gene (*6**,**9**,**10*) and direct sequencing of the VP1 PCR amplicon by using the BigDye Terminator Cycle Sequencing Kit (Applied Biosystems, Foster City, CA, USA). VP1 sequence was compared with VP1 sequences of reference strains available in the National Center for Biotechnology Information (Bethesda, MD, USA). Phylogenetic and molecular evolutionary analyses were conducted by using MEGA4 (www.megasoftware.net). Nucleotide sequences of SAFV strains described were deposited in GenBank under accession nos. HQ668170–HQ668173.

Four (2.7%) of 150 specimens were positive for SAFV (CMH023/2007, CMH038/2007, CMH045/2007, and CMH143/2007). Two of these specimens (CMH023/2007 and CMH038/2007) were obtained in February 2007, one (CMH045/2007) in March 2007, and 1 (CMH143/2007) in November 2007. Co-infections with other viruses were detected in all 4 samples. Two specimens (CMH023/2007and CMH045/2007), were co-infected with noroviruses GII/16 and GII/4 genotypes, respectively. One SAFV-positive sample (CMH038/2007) was co-infected with a group A rotavirus G1P[8] genotype, and another (CMH143/2007) was co-infected with human parechovirus.

All SAFV-positive specimens were further amplified for the VP1 gene to determine their phylogenetic lineages and genetic relationships with other SAFV reference strains. When we used 3 sets of primers used in other studies (*6**,**9**,**10*) for amplification of the VP1 gene, this gene was amplified only by the primer set reported by Itagaki et al. (*10*).

Analysis of partial VP1 sequences (369 nt) of 4 SAFV strains showed that strains CMH023/2007 and CMH143/2007 were highly conserved (nt sequence identities >97%). These 2 SAFV strains were most closely related to the prototype strain of SAFV1 (EF165067) isolated in the United States (nt sequence identity range 87.6%–88.9%) and SAFV strains from China (LZ50419, BCH895, GL311, and GL377) (Figure). In addition, the other 2 SAFVs identified in the present study (CMH038/2007 and CMH045/2007) were identical to each other and closely related to SAFV2 strains from China (BCHU79, BCHU353) and Finland (Finland 2008, FIN08–13B) (nt sequence identity range 94.8%–95.6%). Phylogenetic analysis showed that CMH038/2007 and CMH045/2007 were clustered within the SAFV2 lineage (Figure).

**Figure:**
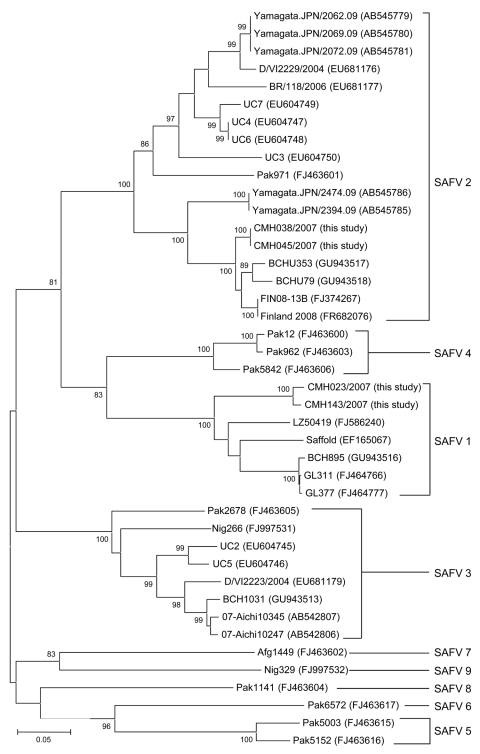
Phylogenetic analysis of the partial nucleotide sequence (369 nt) encoding the viral protein 1 gene of Saffold virus (SAFV) isolated in this study and other reference strains. The tree was generated by using the neighbor-joining method and MEGA4 (www.megasoftware.net). Bootstrap values >80 are indicated for the corresponding nodes on the basis of a resampling analysis of 1,000 replicates. Scale bar indicates nucleotide substitutions per site.

The 4 strains of SAFV were isolated from children with acute gastroenteritis who were co-infected with other viral pathogens (norovirus, group A rotavirus, and human parechovirus). Therefore, we could not determine whether SAFVs identified in this study were associated with acute gastroenteritis. The detection rate for SAFV in children with acute gastroenteritis (2.7%) in our study was consistent with that in a study in Beijing, People’s Republic of China (3.2%) (*9*).

Phylogenetic analysis of the VP1 region demonstrated that 2 SAFV lineages (SAFV1 and SAFV2) were circulating in Chiang Mai, Thailand. Further extensive epidemiologic surveillance of SAFV in other areas may provide a better understanding of the distribution, heterogeneity, and association of SAFV with enteric diseases in humans.
